# The time course of behavioural phase change in the Central American locust *Schistocerca piceifrons*

**DOI:** 10.1242/jeb.244621

**Published:** 2022-12-09

**Authors:** Bert Foquet, Drew W. Little, Jorge Humberto Medina-Durán, Hojun Song

**Affiliations:** ^1^ Department of Entomology, Texas A&M University, College Station, TX 77843-2475, USA; ^2^ School of Biological Sciences, Illinois State University, Normal, IL 61790-4120, USA; ^3^ Department of Biological Sciences, University of Wisconsin - Milwaukee, Milwaukee, WI 53211, USA; ^4^ Behavioral Plasticity Research Institute (BPRI; https://behavioralplasticity.org)

**Keywords:** Phenotypic plasticity, Behaviour, Locust, Polyphenism, Entomology

## Abstract

Locusts exhibit an extreme form of phenotypic plasticity and can exist as two alternative phenotypes, known as solitarious and gregarious phases. These phases, which can transform from one to another depending on local population density, show distinctly different behavioural characteristics. The proximate mechanisms of behavioural phase polyphenism have been well studied in the desert locust *Schistocerca gregaria* and the migratory locust *Locusta migratoria*, and what is known in these species is often treated as a general feature of locusts. However, this approach might be flawed, given that there are approximately 20 locust species that have independently evolved phase polyphenism. Using the Central American locust *Schistocerca piceifrons* as a study system, we characterised the time course of behavioural phase change using standard locust behavioural assays, using both a logistic regression-based model and analyses of separate behavioural variables. We found that for nymphs of *S. piceifrons*, solitarisation was a relatively fast, two-step process, but that gregarisation was a much slower process. Additionally, the density of the gregarisation treatment seemed to have no effect on the rate of phase change. These data are at odds with what we know about the time course of behavioural phase change in *S. gregaria*, suggesting that the mechanisms of locust phase polyphenism in these two species are different and may not be phylogenetically constrained. Our study represents the most in-depth study of behavioural gregarisation and solitarisation in locusts to date.

## Teaser

**Summary:** Behavioural phase transition in the Central American locust differs from that in the desert locust, suggesting that the expression and mechanisms of density-dependent behavioural plasticity in locusts may not be phylogenetically constrained.

## INTRODUCTION

Phenotypic plasticity, the ability of a genotype to produce different phenotypes in response to different environmental conditions, is ubiquitous in nature (Nijhout, 2003; Simpson et al., 2011; West-Eberhard, 2003). Environment-responsive plastic phenotypes include not only easily quantifiable traits, such as morphology, physiology or gene expression patterns, but also behaviour. Behavioural plasticity is more difficult to observe and quantify because one would need to distinguish it from typical behavioural responses to environmental stimuli. Among the organisms that exhibit behavioural plasticity, locusts stand out as a prime model system.

Locusts are a special kind of grasshopper (Orthoptera: Acrididae) that can form devastating migratory swarms through an extreme form of density-dependent phenotypic plasticity (Pener and Simpson, 2009; Uvarov, 1966; West-Eberhard, 2003). Changes in local population density cause solitary individuals to transform into gregarious ones that can migrate en masse (Cullen et al., 2017; Simpson and Sword, 2009; Uvarov, 1966). In addition to behaviour, density affects a myriad of traits, including colour, morphology, neurophysiology, ecology, endocrinology, molecular biology, nutritional physiology, life-history traits and more (Cullen et al., 2017; Pener and Simpson, 2009; Uvarov, 1966). Intriguingly, the expression of these numerous plastic reaction norms is coordinated, and the resulting alternative phenotypes are collectively referred to as ‘solitarious’ and ‘gregarious’ phases (Uvarov, 1966), while the coordinated phenomenon is known as locust phase polyphenism (West-Eberhard, 2003). These two phases are explicitly defined by behavioural traits because behaviour is the first reaction norm that responds to changes in local population density, often within hours (Bouaïchi et al., 1995; Roessingh and Simpson, 1994; Roessingh et al., 1993). Solitarious locusts avoid conspecifics and are relatively inactive, but when the local population increases, they transform into gregarious locusts that are highly active and are attracted to each other, and if the high density persists, they can form large hopper bands or adult swarms that migrate en masse (Cullen et al., 2010, 2012; Foquet et al., 2021; Gray et al., 2009; Guo et al., 2011; Hoste et al., 2002; Pocco et al., 2019; Roessingh et al., 1993; Simpson et al., 1999; Song, 2011; Uvarov, 1966). These stark behavioural differences make it possible to quantify and characterise density-dependent plastic reaction norms in behaviour.

Decades of research on the desert locust *Schistocerca gregaria* and the migratory locust *Locusta migratoria* have established locust phase polyphenism as a tractable system for studying behavioural plasticity (Cullen et al., 2017; Pener and Simpson, 2009). In particular, the behavioural phase transition of the desert locust is so well characterised that the mechanisms known from this species, such as how individual locusts detect changes in density (Anstey et al., 2009; Despland, 2001; Hägele and Simpson, 2000; Roessingh et al., 1998; Rogers et al., 2014, 2003, 2004; Rogers and Ott, 2015; Simpson et al., 2001) or how long it takes for solitarious locusts to change their behaviour to become gregarious and vice versa (Alessi et al., 2014; Anstey et al., 2009; Bouaïchi et al., 1995; Cisse et al., 2015a; Ellis, 1963a,b; Geva et al., 2010; Gillett, 1988; Ott et al., 2012; Roessingh et al., 1998; Roessingh and Simpson, 1994; Rogers et al., 2014), are often treated as a general feature of locusts. However, locust phase polyphenism has evolved multiple times throughout the diversification of grasshoppers, and the 20 known species of locust, which belong to at least four different grasshopper subfamilies, differ markedly from each other in terms of their biology, ecology and habitat preferences (Song, 2011; Song et al., 2017). As such, there is no *a priori* reason to assume that the mechanisms underlying behavioural phase change are identical across all of these locust species, of which many have not been studied in depth. For instance, the migratory locust, the only other species for which the time course of phase change was characterised in detail, shows a very different pattern from what is observed in the desert locust. In the desert locust, solitarious individuals already show clear behavioural changes after only 30 min of crowding, and they become indistinguishable from gregarious individuals after 4 h of crowding (Anstey et al., 2009; Bouaïchi et al., 1995; Cisse et al., 2015a; Ellis, 1963a,b; Geva et al., 2010; Ott et al., 2012; Roessingh and Simpson, 1994; Rogers et al., 2014). However, it takes more than 3 days for gregarious desert locusts to achieve full solitarisation (Alessi et al., 2014; Gillett, 1988; Roessingh and Simpson, 1994). In contrast, the situation seems to be reversed in the migratory locust, which exhibits a fast solitarisation and a very slow gregarisation (Ellis, 1953, 1963a,b; Guo et al., 2011, 2013, 2015; Ma et al., 2011, 2015). These contrasting patterns from these two well-known locust species suggest that the mechanisms of behavioural plasticity are likely different across species, which would require experimental validation.

In the present study, we characterised the temporal process of phase change in the Central American locust *Schistocerca piceifrons* (Walker 1870), a serious pest in Mexico and Central America that causes enormous agricultural damage (Barrientos-Lozano et al., 2021; Cullen et al., 2017). It is a tropical locust species that exhibits classic locust phase polyphenism in terms of behaviour, morphology, colour and gene expression (Barrientos-Lozano et al., 1992; Bredo, 1963; Foquet et al., 2021; Harvey, 1983), but the process of behavioural phase change has not yet been thoroughly characterised in this species (but see Foquet and Song, 2021). Using a standard locust behavioural assay in combination with a logistic regression model, as first developed by Roessingh et al. (1993) for the desert locust and subsequently used to assess behavioural phase change in multiple locust species (Anstey et al., 2009; Bouaïchi et al., 1995; Cullen et al., 2010, 2012; Guo et al., 2011, 2013, 2015; Ma et al., 2015; Ott et al., 2012; Roessingh and Simpson, 1994; Rogers et al., 2014, 2003), we first characterised the time course of behavioural phase change of *S. piceifrons*. We subsequently compared and contrasted the patterns observed in *S. piceifrons* with what has been reported for the desert locust. Although *S. piceifrons* is congeneric to the desert locust, the expression of locust phase polyphenism in this species is considered a result of convergent evolution (Song et al., 2017), and therefore, we hypothesised that the specific time course of behavioural phase transition would be different between the two locust species.

## MATERIALS AND METHODS

### Lab colony and rearing regime

The lab colony of *S. piceifrons*, as well as its rearing regimes, was previously described by Foquet and Song (2021). It originated from an outbreak population in Yucatán, Mexico, in 2015, and was imported under a USDA permit (USDA APHIS PPQ P526P-15-03851) and kept in a USDA-approved quarantine facility in the Department of Entomology at Texas A&M University. The insects were kept at 30°C with a 12 h:12 h light:dark cycle, and were fed romaine lettuce and wheat bran daily. The relative humidity was kept at 50%. Locusts were reared under crowded conditions (200+ individuals) in large plastic containers (40.64×34.29×52.07 cm), of which one side and the top was replaced with mesh. The bottom of the cage contained wood shavings for nymphs, or a larger mesh for adults. Heat lamps positioned above the cages allowed locusts to thermoregulate during daytime. As the population density is the main environmental stimulus for locust phase polyphenism, both phases can easily be induced in the laboratory (Foquet et al., 2021). To induce the solitarious phase, hatchlings were isolated into individual plastic cages (10.16×10.16×25.4 cm) with three non-transparent sides and connected to a positive filtered airflow, so that the nymphs were physically, visually and chemically isolated from one other. To induce the gregarious phase, locusts were kept with approximately 250 individuals in the large cage (40.64×34.29×52.07 cm) described above. All experiments were performed 3 to 5 days after the nymphs moulted to their final nymphal instar, and food was given *ad libitum* for the duration of the experiments.

### Behavioural assays

Behavioural assays were performed using a Roessingh-type rectangular arena (57×31×11 cm) with an empty non-stimulus chamber on one end and a stimulus chamber containing 30 last-instar nymphs on the other end (Foquet et al., 2021; Roessingh et al., 1993). For each assay, the test subject was placed into a blackened 50 ml Falcon tube for 2 min before introducing it into the arena. Each locust was filmed for 12 min at a frame rate of 30 frames s^−1^ with a Panasonic HDC-TM900 (704×480 pixels, 29.97 frames s^−1^) or a Basler acA1300–60gc (1280×1024 pixels, 60 frames s^−1^) camera, and EthoVision XT 12 (Noldus Information Technology Inc., Leesburg, VA, USA) software was used to video-track locusts. The arena was divided into three equal zones: a stimulus zone (the third adjacent to the stimulus chamber), a non-stimulus zone (the third adjacent to the non-stimulus chamber) and a neutral zone (the central third). Additionally, the walls were designated as a wall zone, which overlapped with the other zones. We used ‘differencing’ as a detection method, and the subject colour was set as ‘darker and lighter’ than the background. The track of each behavioural assay was manually inspected and corrected where necessary, after which they were smoothened using a minimum distance moved filter of 0.2 cm (Foquet et al., 2021). A set of 11 variables was exported as described before (Kilpatrick et al., 2019), including five activity-related and six attraction-related variables. The activity-related variables were distance moved (cm), movement (s), velocity (cm s^−1^), rotation frequency and wall climbing (s), and the attraction-related variables were stimulus zone time (s), neutral zone time (s), non-stimulus zone time (s), stimulus wall time (s), mean distance to stimulus (cm) and final distance to stimulus (cm). The velocity was calculated by dividing the distance moved by the movement, rather than directly exporting it from EthoVision.

### Logistic regression model

To summarise these behavioural variables into a simplified measure of the behavioural phase state, we developed a binary logistic regression model in R version 4.1.2 (https://www.r-project.org/), following standard procedure in locust phase research (see e.g. Cullen et al., 2017; Rogers et al., 2014). Behavioural data were obtained from 137 isolated-reared individuals and 109 crowd-reared individuals (Table S1), which were partially published in Foquet et al. (2021). These were randomly split into a training set (80% of individuals) and a test set (20% of individuals). Using the training set, we used stepwise logistic regression to select predictor variables from the 11 behavioural variables. Variables were added or removed until the Akaike’s information criterion (AIC) did not significantly change between steps. The first model retained four behavioural variables: movement, stimulus wall time, rotation frequency and neutral zone time. In a second and final run, variables that were not expected to contain biologically relevant information (neutral zone time; see Foquet et al., 2021) and variables that were highly correlated to one of the variables included in this first model (e.g. distance moved) were removed from the dataset. This time, the optimally fitting logistic regression model retained only three variables: movement, stimulus wall time and rotation frequency. A bootstrap with 1000 replications was performed on this model, after which the following equation was obtained: γ=−4.6707993476+0.0136989875×movement+0.0041056885×stimulus wall time+0.0582089976×rotation frequency (Table S2). Subsequently, *P*_greg_ [*P*_greg_=e^γ^/(1+e^γ^)] was calculated as a linear predictor of the phase state of a test subject that varies between 0 (fully solitarious) to 1 (fully gregarious) (Roessingh and Simpson, 1994; Roessingh et al., 1993; Rogers et al., 2014). Using a threshold of 0.5, *P*_greg_ correctly identified 91.8% of the training set (93.8% of isolated-reared individuals and 88.9% of crowd-reared individuals) and 85.7% of the test set (88.0% of isolated-reared nymphs and 83.3% of crowd-reared nymphs), for an overall identification rate of 90.5%.

### Time-course experimental design

For the time course of solitarisation, crowd-reared nymphs were placed into the plastic cages used for inducing the solitarious phase, and were kept singly in these cages for either 1, 2, 4, 8, 12, 24 or 48 h, after which a behavioural assay was performed on each insect. For the time course of gregarisation, isolated-reared nymphs were placed into a small cage (30.48×35.56×50.8 cm) containing 50 crowd-reared conspecifics, and a behavioural assay was performed after 1, 2, 4, 8, 12, 24 or 48 h. We additionally tested the effect of crowding density in behavioural gregarisation by keeping the duration of crowding constant at 2 h but by varying the number of gregarious nymphs (25, 50, 100 and 150 nymphs) in the cage. For each tested time point or density, 30 different individuals were tested, with each individual tested only once. Each behavioural assay was performed in the afternoon to attempt to reduce any temporal effects on behaviour. The effect of time or density on either *P*_greg_ values or behavioural variables was analysed with a Kruskal–Wallis test in R 4.1.2, and for *post hoc* pairwise comparisons between different time points, a pairwise Wilcoxon test with Benjamini–Hochberg correction was used in R 4.1.2. All behavioral data can be found in Table S1.

## RESULTS

**Fig. 1.**
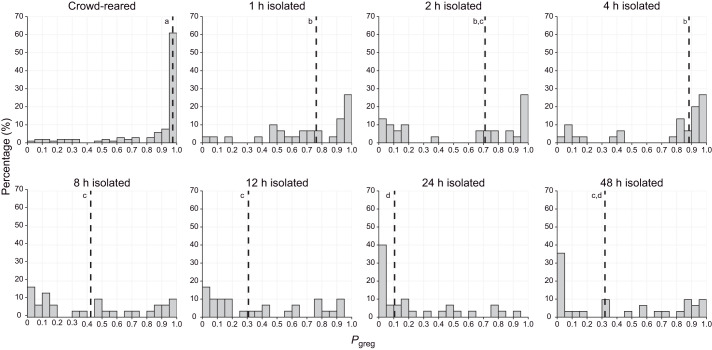
**Time course of solitarisation.** Frequency histograms showing the percentage of last-instar crowd-reared nymphs of *Schistocerca piceifrons* that fall into different categories of behavioural phase state (*P*_greg_) after being isolated for varying amounts of time. The number of hours reflects the duration of the isolation treatment, and the graph labeled ‘crowd-reared’ represents the control group. Dashed lines indicate the median values of *P*_greg_ for each group. Graphs showing the same letter were not significantly different (pairwise Wilcoxon tests, *P*<0.05). *n*=30 for each graph, except for crowd-reared (*n*=109).

When we isolated crowd-reared nymphs for various durations, we observed a significant effect of treatment time on *P*_greg_ (Kruskal–Wallis chi-squared=104.31, *P*<2.2×10^−16^; Fig. 1). All isolation treatments, including the 1 h isolation treatment, differed significantly from the crowd-reared control, and isolated locusts behaved more solitariously as time progressed in several distinct stages (Fig. 1). A rapid, but partial solitarisation in the first hour was followed by a slower period between 1 and 4 h after the onset of isolation, with a median *P*_greg_ still larger than 0.5 after 4 h (Fig. 1). From 8 h of isolation onwards, the majority of the locusts were behaviourally categorised as solitarious, and *P*_greg_ at 8 h of isolation treatment was significantly different from *P*_greg_ after 1 or 4 h of isolation (Fig. 1). After 24 h, just under half of the tested locusts had a *P*_greg_≤0.1, and the median *P*_greg_ significantly differed from all other time points. Even though the median *P*_greg_ became slightly higher again after 48 h of isolation treatment, it was still well below 0.5.

**Fig. 2.**
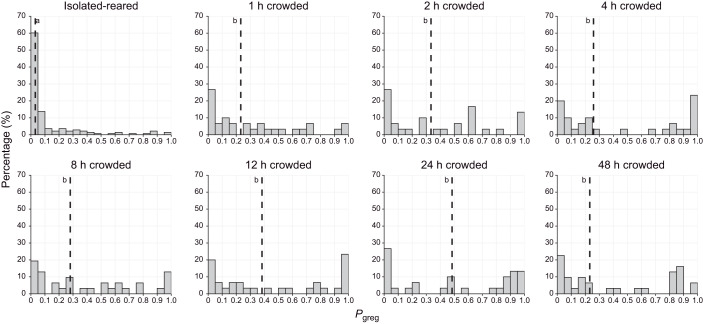
**Time course of gregarisation.** Frequency histograms showing the percentage of last-instar isolated-reared nymphs of *S. piceifrons* that fall into different categories of behavioural phase state (*P*_greg_) after being crowded for varying amounts of time. The crowding treatment consisted of being placed in a small cage with 50 crowd-reared last-instar nymphs. The number of hours reflects the duration of the isolation treatment, and the graph labeled ‘isolated-reared’ represents the control group. Dashed lines indicate the median values of *P*_greg_ for each group. Graphs showing the same letter were not significantly different (pairwise Wilcoxon tests, *P*<0.05). *n*=30 for each graph, except for isolated-reared (*n*=137).

When we induced gregarisation by crowding isolated-reared nymphs for various durations, we again observed a significant effect of treatment time on *P*_greg_ (Kruskal–Wallis chi-squared=72.094, *P=*5.571×10^−13^; Fig. 2). Similarly to the time course of solitarisation, we observed that crowded locusts already differed significantly from the isolated-reared control treatment within 1 h of crowding (pairwise Wilcoxon test, *P*=0.00011; Fig. 2). However, we did not observe any evidence of further behavioural gregarisation after the 1 h time point. Indeed, the median *P*_greg_ never became larger than 0.5, and no more significant differences in *P*_greg_ were observed between the other time points in the gregarisation time course (Fig. 2). Even after 48 h of crowding, isolated-reared locusts did not fully gregarise, and more than 50% of the locusts at this time point were still classified as gregarious.

**Fig. 3.**
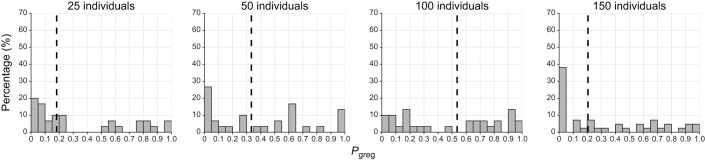
**The effect of density on gregarisation.** Frequency histograms showing the percentage of last-instar isolated-reared nymphs of *S. piceifrons* that fall into different categories of behavioral phase state (*P*_greg_) after being crowded in varying densities for 2 h. Isolated-reared nymphs were placed in small cages containing 25, 50, 100 or 150 crowd-reared individuals. Dashed lines indicate the median values of *P*_greg_ for each group. No significant differences were found with a Kruskal–Wallis chi-squared test. *n*=30 for each graph.

Because the lack of full gregarisation could be an artefact of the density used for the crowding treatment, we subsequently tested the effect of different densities while keeping the crowding duration constant at 2 h. We did not find any statistically significant effect of density on *P*_greg_ (Kruskal–Wallis chi-squared=3.506, d.f.=3, *P*=0.32), even though there was a rather large extent of variation in the median *P*_greg_ (Fig. 3). In other words, we found no evidence for an effect of the crowding density on behavioural gregarisation in the first 2 h of crowding.

**Fig. 4.**
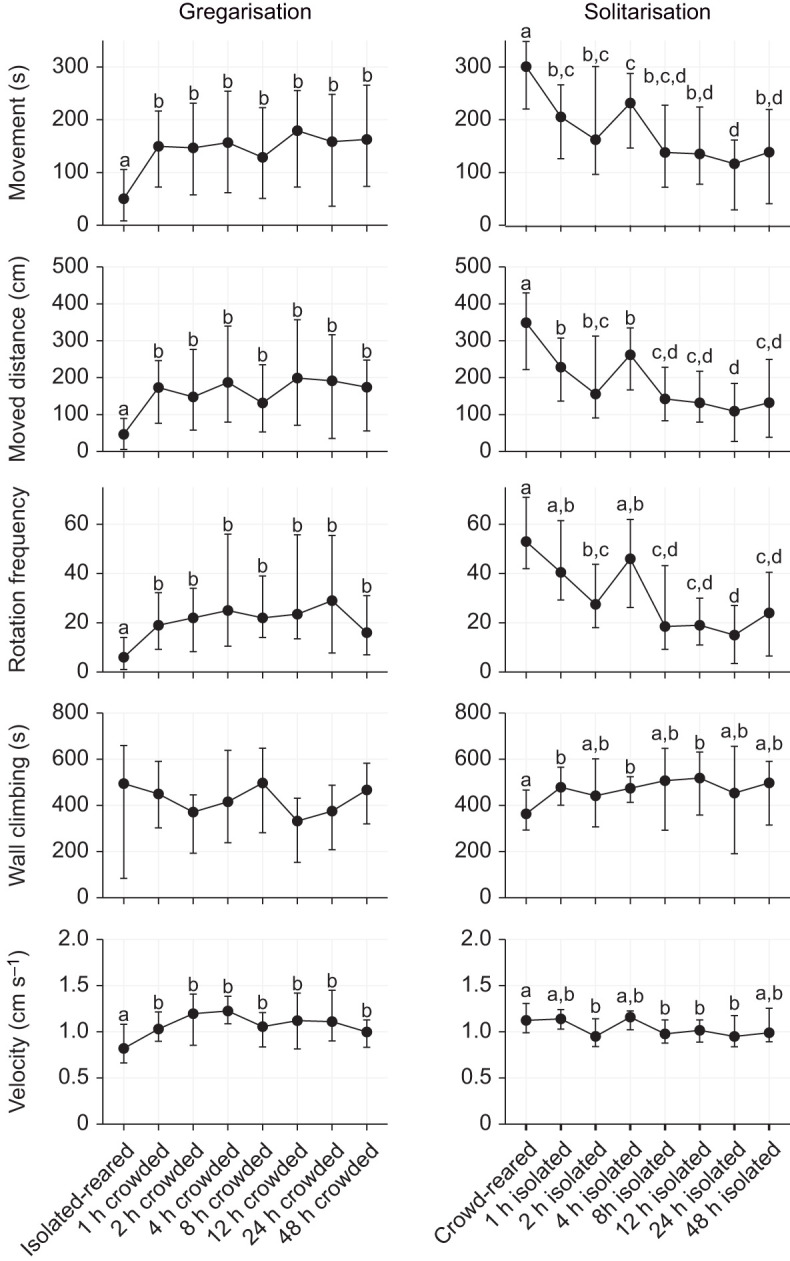
**Activity-related variables change at similar rates during crowding and isolation.** Graphs show the changes in the five activity-related behavioural variables that were used in generating *P*_greg_ as an effect of time of solitarisation (right) or time of gregarisation (left). Graphs show the median value for each group (circle) and error bars represent the 75th percentile of observations. Statistical significance was analysed with a Kruskal–Wallis test followed by paired Wilcoxon tests with a Benjamini–Hochberg correction as *post hoc* tests. Data points showing the same letter were not significantly different for the *post hoc* test. Graphs without letters had no significant effect of time since onset of treatment, as shown by the Kruskal–Wallis test. *n*=30 for each graph, except for crowd-reared (*n*=109) and isolated-reared (*n*=137).

**Fig. 5.**
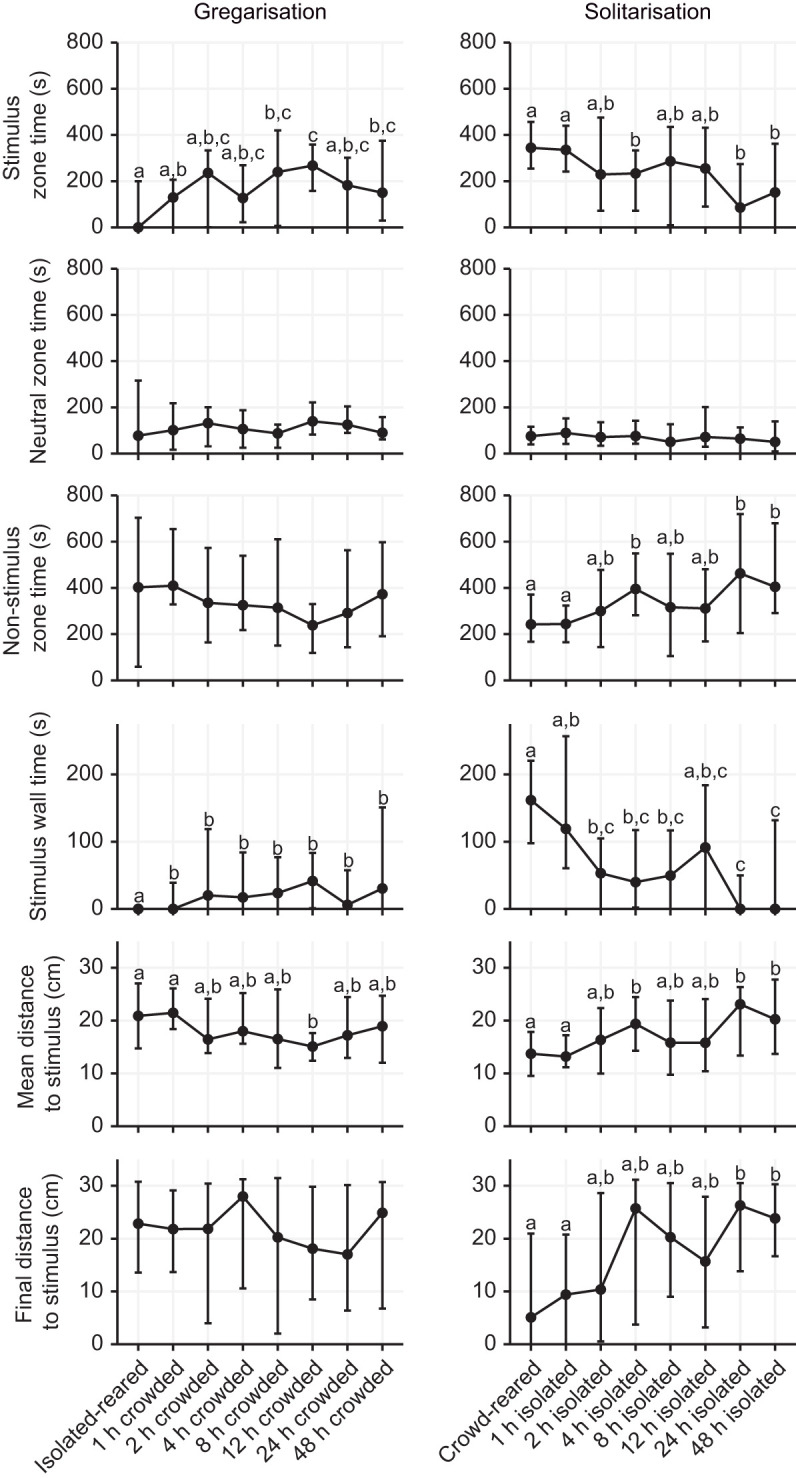
**Attraction-related variables change at similar rates during crowding and isolation.** Graphs show the changes in the six attraction-related behavioural variables that were used in generating the *P*_greg_ as an effect of time of solitarisation (right) or time of gregarisation (left). Graphs show the median value for each group (circle) and error bars represent the 75th percentile of observations. Statistical significance was analysed with a Kruskal–Wallis test followed by paired Wilcoxon tests with a Benjamini–Hochberg correction as *post hoc* tests. Data points showing the same letter were not significantly different for the *post hoc* test. Graphs without letters had no significant effect of time since onset of treatment, as shown by the Kruskal–Wallis test. *n*=30 for each graph, except for crowd-reared (*n*=109) and isolated-reared (*n*=137).

We subsequently sought to investigate whether the different behavioural variables, including the ones used to build the logistic regression model, changed simultaneously or whether they showed independent time courses (Figs 4 and 5). We found that most behavioural variables changed in a very similar fashion as *P*_greg_. Gregarisation was only partially completed, and significant changes in behaviour occurred within the first 2 h of the crowding treatment (Figs 4 and 5; Table S3). In contrast, solitarisation was a more dynamic process that changed more gradually throughout the experiment (Figs 4 and 5; Table S3). Interestingly, for most variables, the endpoint of the solitarisation time course still seemed to differ from the isolated-reared control. Some behavioural variables, such as neutral zone time and wall climbing, seemed to be mostly unaffected by the crowding and isolation treatments, and also showed similar values for both crowd-reared and isolated-reared controls. Notably, the three variables that were used to build the logistic regression model (stimulus wall time, movement and rotation frequency) exhibited concerted changes (Figs 4 and 5; Table S3). We found no significant differences for any behavioural traits when varying the density during the 2 h crowding treatment, with the exception of wall climbing, which was significantly lower at a rearing density of 50 (Fig. S1, Table S3).

## DISCUSSION

Our data showed that behavioural solitarisation is a relatively fast process in last-instar nymphs of *S. piceifrons* (Figs 1 and 4), with a fast initial change in the first hour, after which locust behaviour gradually changes until it reaches solitarious-like values after 24 h. In contrast, behavioural gregarisation is much slower (Figs 2 and 4), and is characterised by a fast partial change in the first hour, followed by a plateau phase of at least 2 days in which locusts are generally still behaving more like solitarious locusts. The level of this plateau phase seems to be independent of locust density (Fig. 3). As expected, the behavioural phase change reported here is quite different from what has been consistently reported in the congeneric desert locust.

A large number of studies in the desert locust have shown that solitarious desert locusts become almost completely gregarised within 4 h of crowding, while the opposite process takes multiple days to complete, even though the initial behavioural changes of the solitarisation process can occur within an hour (Alessi et al., 2014; Anstey et al., 2009; Bouaïchi et al., 1995; Cisse et al., 2015a; Ellis, 1963a,b; Geva et al., 2010; Gillett, 1988; Ott et al., 2012; Roessingh and Simpson, 1994; Rogers et al., 2014). These data are consistent throughout a large number of studies in the desert locust, even though they were obtained using multiple behavioural assays and by measuring different behavioural variables. This is a testament to the robustness of behavioural phase change within the desert locust, and allows us to compare behavioural phase change with another species, even if the experimental conditions and measured behavioural traits differ (Cullen et al., 2017, 2012; Rogers et al., 2014).

Although for both *S. piceifrons* and *S. gregaria* the initial behavioural changes for both solitarisation and gregarisation occur rapidly (<1 h), the time needed to complete the processes of gregarisation and solitarisation is very different between the two species. The desert locust is adapted to xeric habitats where food is often scarce, and it is currently thought that suitable habitat for desert locust breeding commonly emerges after rains. Owing to its rapid gregarisation, the resulting increase in population density induces a shift from avoidance to attraction behaviour. These behavioural changes subsequently further increase local population densities, leading to even more density-related phenotypic changes along the way (Bouaïchi and Simpson, 2003; Despland et al., 2000; Roessingh et al., 1993; Uvarov, 1966). The swift gregarisation in *S. gregaria* enables them to quickly take advantage of changes in habitat and food availability. Additionally, gregarious locusts that get separated from the swarm remain active for a long time, enabling them to easily rejoin the swarm. Overall, this pattern suggests that for the desert locust, it is advantageous for solitarious nymphs to join a hopper band if possible. This notion is further supported by the observation that the Australian plague locust *Chortoicetes terminifera*, another locust species that inhabits arid areas and exhibits a fast gregarisation (Cullen et al., 2010), was shown to survive longer on average in a group than on their own under conditions of food scarcity (Hansen et al., 2011). In contrast, *S. piceifrons* is a tropical locust that commonly swarms in pastures with a regrowth of woody shrubs and other areas with a recent change in land use (Harvey, 1981). Here, they have access to a much more predictable access to food, often including the grass *Panicum maximum* which is a preferred host plant (Poot-Pech et al., 2018). It might also be a more advantageous strategy for the last instar solitarious nymph of *S. piceifrons* to hide away in the abundant vegetation from potential predators, rather than to join a passing hopper band, especially because at that point of its life it would be maladapted to the gregarious lifestyle. As such, it has to be further elucidated whether the initial changes seen in the first hour of behavioural gregarisation in *S. piceifrons* are truly the first step of the gregarisation process, or are due to habituation to the new, more crowded environment, as described in *S. gregaria* (Geva et al., 2010). Similarly, gregarious locusts that become separated from the swarm can easily disappear into the vegetation, where food is still plentiful and they are safe from predators. Indeed, an earlier study shows that locusts can use thorny bushes such as *Pisonia aculeata* for shelter (Poot-Pech et al., 2016). Interestingly, there are several parallels between *S. piceifrons* and *L. migratoria*, which also exhibit a fast solitarisation and a slow gregarisation (Ellis, 1953, 1963a,b; Guo et al., 2011, 2013, 2015; Ma et al., 2011, 2015), and occur in grassland habitats where there is also a lot of cover for solitarious locusts. This suggests that the evolution of the time courses of gregarisation and solitarisation might be, at least to an extent, influenced by the habitat preferences. This indicates that the expression and mechanisms of behavioural plasticity can differ between closely related species and is shaped more by local adaptation than by shared ancestry. Other examples exist that show that the mechanisms of phenotypic plasticity differ between closely related species. For instance, plasticity in eyespots in closely related nymphalid butterflies varies in whether it is continuous or discrete, and in what direction it responds to temperature, even though the two extreme phenotypes are very similar between all species (van Bergen et al., 2017), and two congeneric species of tadpoles (*Scaphiopus hammondii* and *S. couchii*) speed up metamorphosis in response to diminishing water volumes, but detect changing water levels by different cues (Denver et al., 1998; Newman, 1994).

Finally, our data can also answer some long-standing questions in locust research. For instance, attraction-related and activity-related behaviours seem to change at the same rate during behavioural phase change in *S. piceifrons* (Figs 4 and 5), as also seems to be the case in *S. gregaria* (Rogers et al., 2014) and in contrast to what was suggested by Tanaka and Nishide (2013). However, Guo et al. (2011) reported differences in the rate at which attraction-related and activity-related variables changed for both gregarisation and solitarisation in *L. migratoria*, suggesting that this too might differ between the different locust species. Additionally, our study represents the first time that the effect of density during the crowding treatment was tested (but see Cisse et al., 2015b and Heifetz et al., 1996 for the long-term effect of rearing density on behaviour). The density used in our gregarisation experiment equals approximately 100 individuals m^−2^, which is well below the high densities found in active hopper bands (Barrientos-Lozano et al., 2021). However, increasing or decreasing the density did not significantly alter the gregarisation state of the locusts, and the lowest median *P*_greg_ was in fact observed at the highest rearing density. Overall, our finding seems to agree with the earlier findings in the desert locust, where only a few individuals are needed to induce gregarisation (Cisse et al., 2015b; Heifetz et al., 1996).

Our study represents the most in-depth and complete study of behavioural gregarisation and solitarisation in locusts to date, by studying both *P*_greg_, a composite value giving the probability of gregariousness, and separate behavioural variables. It supports the notion that the expression and mechanisms of density-dependent behavioural plasticity in locusts may not be phylogenetically constrained, and shows that we cannot simply extrapolate what is known from one locust species to another. As such, an increased sampling of locust species is much needed, in order to obtain a better understanding of behavioural phase change in locusts. Finally, our study shows that different locust species in the genus *Schistocerca* evolved highly similar phenotypes in a different manner, which, together with the existence of closely related non-swarming species, makes this genus an excellent model clade for the study of the evolution of phenotypic plasticity.

## Supplementary Material

10.1242/jexbio.244621_sup1 Supplementary information
