# Supplementary material for: The time course of behavioural phase change in the Central American locust Schistocerca piceifrons
Source: J Exp Biol. 2022 Dec 9;225(23):jeb244621. doi: 10.1242/jeb.244621 (PMC9789408; doi:10.1242/jeb.244621)
Supplement: Supplementary information [file jexbio-225-244621-s1.pdf]

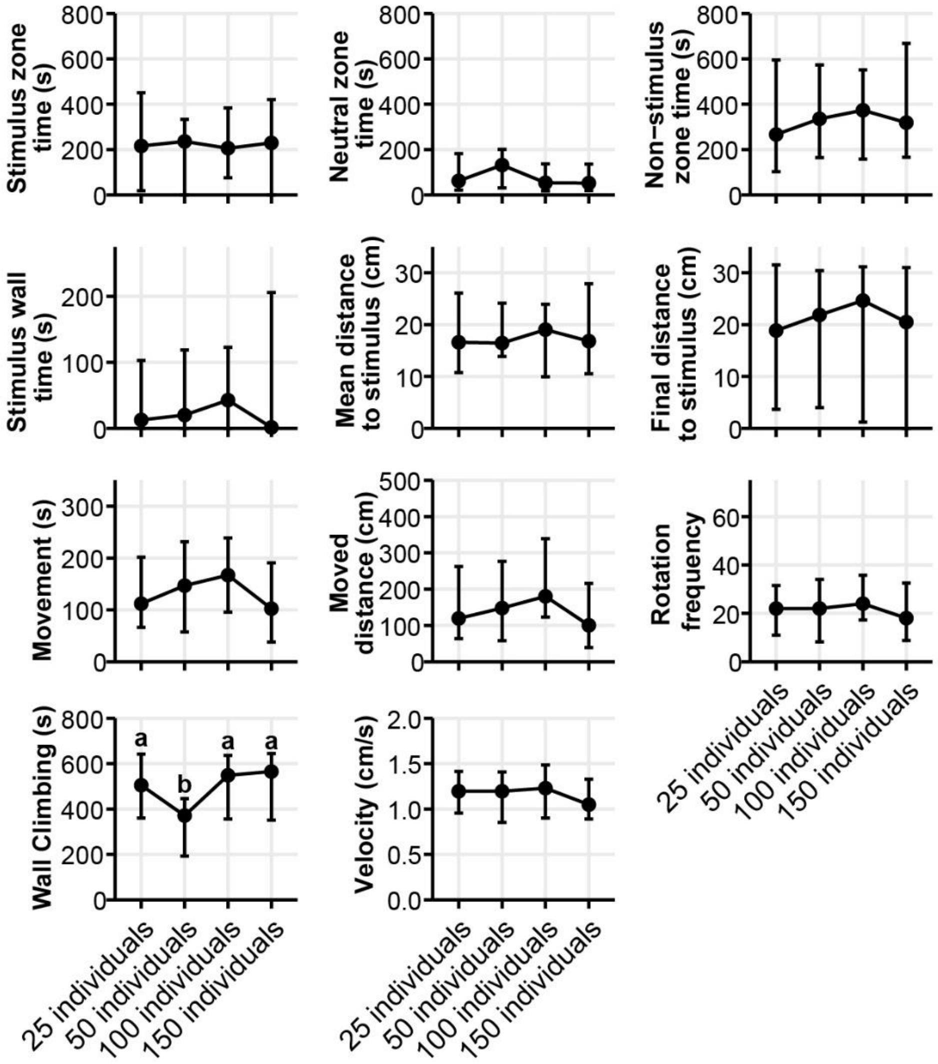

Fig. S1. Individual behavioural variables do not change with changing crowding density. Graphs showing the changes in behavioural variables as an effect of different densities used during a two hour crowding treatment. Graphs are showing the median value for each group and error bars represent the 75th percentile of observations. Statistical significance was analysed with a Kruskal-Wallis test followed by paired Wilcoxon-test with a Benjamini-Hochberg correction as *post-hoc* tests, and significance of the post-hoc test was shown with a compact letter display above the graph. Graphs without a letter display represent variables for which the Kruskal-Wallis test was not significantly different.

Journal of Experimental Biology • Supplementary information

Table S1. Relevant behavioral data of time-courses of gregarization and solitarization, and effect of density.

Click here to download Table S1

Table S2. Bootstrapping output of logistic regression model.

Variable	Original	Change after bootstrap	Standard error	lower 95% conf. interval	upper 95% conf. interval
	-4.460730342	-0.210069006	0.729391356	-5.733	-3.318
Movement (s)	0.013321239	0.000377749	0.004287541	0.0051	0.0281
Stimulus wall time (s)	0.003881505	0.000224184	0.002067104	0.0000	0.0081
Rotation frequency	0.052695775	0.005513223	0.021804809	0.0161	0.0943

Journal of Experimental Biology • Supplementary information

Table S3. Nonparametric analysis of the effect of solitarisation, gregarisation and density course on locust behaviour. Statistical analysis was performed with a Kruskal-Wallis analysis in R 4.1.2. Significant values are shown in bold.

Variable	Experiment	Kruskal-Wallis chi-squared	df	p value
Movement (s)	Solitarisation	81.025	7	**8.51E-15**
	Gregarisation	61.249	7	**8.50E-11**
	Density	5.7404	3	0.1249
Rotation Frequency	Solitarisation	96.279	7	**< 2.2e-16**
	Gregarisation	68.241	7	**3.35E-12**
	Density	2.1439	3	0.5431
Stimulus wall time (s)	Solitarisation	50.801	7	**1.01E-08**
	Gregarisation	47.473	7	**4.51E-08**
	Density	1.0725	3	0.7837
Distance Moved (cm)	Solitarisation	80.424	7	**1.13E-14**
	Gregarisation	68.739	7	**2.66E-12**
	Density	6.9369	3	0.07394
Velocity (cm/s)	Solitarisation	27.684	7	**0.0002508**
	Gregarisation	41.056	7	**7.90E-07**
	Density	1.5927	3	0.661
Wall climbing (s)	Solitarisation	18.799	7	**0.008839**
	Gregarisation	11.619	7	0.1138
	Density	13.134	3	**0.004355**
Stimulus zone time (s)	Solitarisation	40.523	7	**9.99E-07**
	Gregarisation	30.941	7	**6.38E-05**
	Density	1.0213	3	0.7961
Non-stimulus zone time (s)	Solitarisation	26.336	7	**0.0004386**
	Gregarisation	12.882	7	0.07504
	Density	0.72319	3	0.8677
Neutral zone time (s)	Solitarisation	5.4025	7	0.611
	Gregarisation	7.5805	7	0.371
	Density	3.6758	3	0.2987
Mean distance to stimulus (cm)	Solitarisation	32.528	7	**3.24E-05**
	Gregarisation	17.065	7	**0.01698**
	Density	0.36001	3	0.9484
Final distance to stimulus (cm)	Solitarisation	23.804	7	**0.001233**
	Gregarisation	4.9147	7	0.6704
	Density	0.72977	3	0.8662
